# Silencing, Positive Selection and Parallel Evolution: Busy History of Primate Cytochromes *c*

**DOI:** 10.1371/journal.pone.0026269

**Published:** 2011-10-18

**Authors:** Denis Pierron, Juan C. Opazo, Margit Heiske, Zack Papper, Monica Uddin, Gopi Chand, Derek E. Wildman, Roberto Romero, Morris Goodman, Lawrence I. Grossman

**Affiliations:** 1 Center for Molecular Medicine and Genetics, Wayne State University, School of Medicine, Detroit, Michigan, United States of America; 2 Perinatology Research Branch, National Institute of Child Health and Development, National Institutes of Health, Bethesda, Maryland and Detroit, Michigan, United States of America; 3 Instituto de Ecologia y Evolucion, Facultad de Ciencias, Universidad Austral de Chile, Valdivia, Chile; 4 Laboratoire de Physiopathologie Mitochondriale, INSERM, Université Victor Segalen Bordeaux 2, Bordeaux, France; 5 School of Public Health, The University of Michigan, Ann Arbor, Michigan, United States of America; 6 Department Of Obstetrics and Gynecology, Wayne State University, School of Medicine, Detroit, Michigan, United States of America; 7 Department of Anatomy and Cell Biology, Wayne State University, School of Medicine, Detroit, Michigan, United States of America; British Columbia Centre for Excellence in HIV/AIDS, Canada

## Abstract

Cytochrome *c* (cyt *c*) participates in two crucial cellular processes, energy production and apoptosis, and unsurprisingly is a highly conserved protein. However, previous studies have reported for the primate lineage (i) loss of the paralogous testis isoform, (ii) an acceleration and then a deceleration of the amino acid replacement rate of the cyt *c* somatic isoform, and (iii) atypical biochemical behavior of human cyt *c*. To gain insight into the cause of these major evolutionary events, we have retraced the history of cyt *c* loci among primates. For testis cyt *c*, all primate sequences examined carry the same nonsense mutation, which suggests that silencing occurred before the primates diversified. For somatic cyt *c*, maximum parsimony, maximum likelihood, and Bayesian phylogenetic analyses yielded the same tree topology. The evolutionary analyses show that a fast accumulation of non-synonymous mutations (suggesting positive selection) occurred specifically on the anthropoid lineage root and then continued in parallel on the early catarrhini and platyrrhini stems. Analysis of evolutionary changes using the 3D structure suggests they are focused on the respiratory chain rather than on apoptosis or other cyt *c* functions. In agreement with previous biochemical studies, our results suggest that silencing of the cyt *c* testis isoform could be linked with the decrease of primate reproduction rate. Finally, the evolution of cyt *c* in the two sister anthropoid groups leads us to propose that somatic cyt *c* evolution may be related both to COX evolution and to the convergent brain and body mass enlargement in these two anthropoid clades.

## Introduction

Cytochrome *c* (cyt *c*), a protein with 104 amino acids, contains at least five known functions: a) transfer of electrons from complex III to complex IV in the electron transport chain, b) initiation of apoptosis by forming a complex with Apaf-1 to activate caspase-9, c) acting as a cardiolipin oxygenase at an earlier step in promoting apoptosis, d) acting as an electron donor to p66^Shc^ to mediate mitochondrial apoptosis, and e) acting as an electron acceptor in the translocation of mitochondrial intermembrane space proteins with disulfide bonds [1].

Both this functional density and the central role of these functions for life help to explain why cyt *c* evolves slowly [2], [3] among eukaryotes. For example, yeast and mammals share about 45% amino acid identity; and, within mammals, mouse (*Eutheria*) and platypus (*Prototheria*) share 95.3% amino acid identity. The two most studied eutherian somatic cyt *c*s, mouse and cow, share 97.2% amino acid identity. Despite this overall sequence conservation, human cyt *c* presents several atypical properties compared to other related mammals: (i) human (and other primates) has only a single cyt *c* gene ubiquitously expressed in all tissues whereas mouse has one testis isoform and one somatic isoform [4]; (ii) human and mouse somatic cyt *c* sequences exhibit 9 amino acid differences (93.4% identity) due to a relatively fast amino acid replacement rate during human descent from the LCA of Primates [5]; and (iii) biochemical analyses have pointed to a higher affinity of human cyt *c* with electron transport chain complexes [6], [7] and a pKa value for the alkaline transition higher than those for horse and yeast [8].

In the past decade our group has shown a rapid evolution of cytochrome *c* oxidase in primates [9]. We have investigated here whether the atypical properties of human somatic cyt *c* are due to (i) the silencing of cyt *c* testis isoform, (ii) coevolution with cytochrome *c* oxidase, or (iii) a co-evolutionary step involving both silencing of cyt *c* testis isoform and evolution of cytochrome *c* oxidase.

To address these questions, we have (i) performed a detailed phylogenetic study of somatic cyt *c* sequences across the primates in order to gain insight into the tempo of accumulation of amino acid differences between human and mouse; (ii) investigated the somatic cyt c functions affected by these mutations in a structural context; and (iii) studied the testis cyt *c* gene locus across vertebrates and primates in order to understand the scenario of its appearance and silencing.

This work has also allowed us to study the phylogenetic relationships among primates and the divergence times based on somatic cytochrome *c* divergence.

## Results

### Phylogenetic relationships among primates estimated from cytochrome *c* divergence

**Figure 1:**
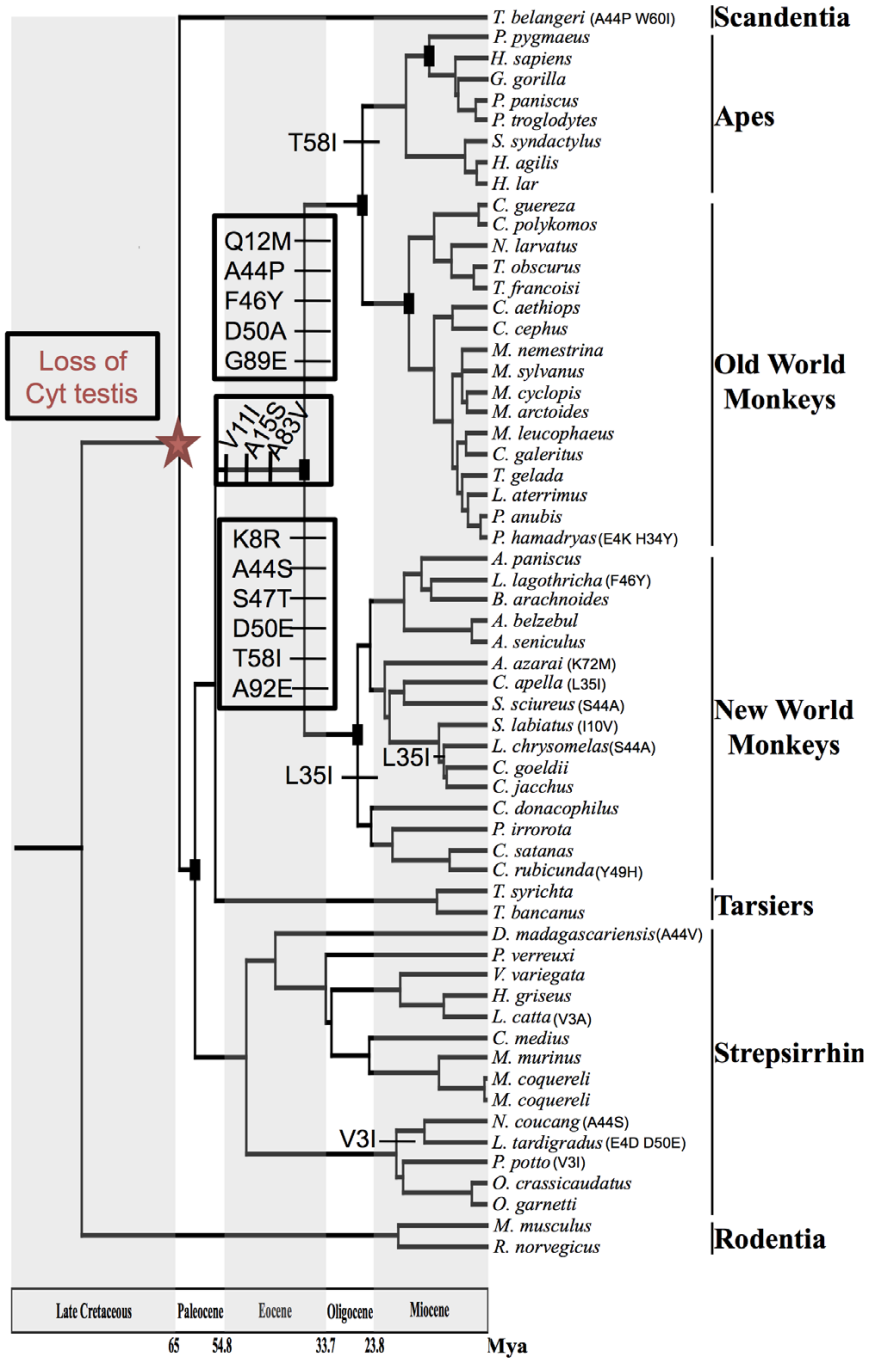
Phylogenetic relationships among primates estimated from cytochrome *c* sequence. The same phylogenetic tree was obtained using (separately) maximum likelihood, maximum parsimony and Bayesian approaches. Divergence times were estimated by the Bayesian local molecular clock approach. Only non-synonymous mutation events of internal branches are shown.

Using maximum likelihood, maximum parsimony, and Bayesian approaches on the cyt *c* somatic gene sequence (CYCS, GeneBank Accession numbers: JF919224-JF919284), we estimated phylogenetic relationships among 56 primate and 4 non-primate species. Furthermore, using a Bayesian local molecular clock approach, we also estimated divergence times for the whole group. All phylogenetic analyses converged to the same tree topology (Figure 1). The tree topology depicts a first split that separated Rodentia from Scandentia and Primates, then a split that separated Scandentia from Primates (Figure 1). Among Primates, the major crown groups were recovered with high bootstrap support. Additionally, all major groups (apes, Old World monkeys, New World monkeys, tarsiers and strepsirrhines) were recovered with maximal branch support. Among apes, small (*e.g.*, gibbons and siamangs) and large (*e.g.*, orangutans, gorillas, chimpanzees, humans) bodied apes were recovered as monophyletic groups with high branch support. Among Old World monkeys, colobini (*e.g.*, colobus monkeys) and cercopithecinae (*e.g.*, baboons and macaques) were recovered as monophyletic groups with maximal branch support. In the first group, both Asian and African clades were also recovered with high branch support. Among New World monkeys (*e.g.*, capuchin monkeys, marmosets, titi monkeys) our results support three platyrrhine families with moderate branch support. Among New World monkey, according to our results Pithecidae is a sister group of a clade containing Cebidae and Atelidae, and this grouping was supported with moderate to high branch support. Among strepsirrhines, lemuriformes (*e.g.*, lemurs and sifakas) and lorisiformes (lorises and galagos) were recovered as monophyletic groups with high bootstrap support (Figure S1).

### Divergence times

Using a Bayesian relaxed molecular clock approach, we estimated divergence times for the primates and crown group. Consistent with previous evaluations we estimated the origin of primates as a total and crown group in the early Paleocene, 67.34±5.25 and 63.38±5.17 Mya (± SD), respectively (Figure 1, Table S1). The origin of strepsirrhines as a crown group was estimated in the early Eocene, 52.25±.05 Mya, whereas the origin as a crown group of the lemuriformes and lorisiformes was estimated in the middle Eocene, 46.28±3.33 Mya, and in the early Miocene, 19.36±1.45 Mya, respectively. The origin of the haplorrhine clade was dated in the late Paleocene, 59.00±4.49 Mya.

### Primate somatic cytochrome *c* evolution

The study of amino acid evolution using Maximum parsimony on the primate lineages shows the presence of many “living fossil” cyt *c* sequences, *i.e.*, proteins that have exactly the same amino-acid sequence as the ancestral primate cyt c (Figure 1). However, we inferred 3 amino acid replacements occurred on the anthropoid stem, *i.e.*, before the radiation of New World monkeys, Old World monkeys, and apes. Thereafter, rapid accumulation of replacements continued in parallel in the early platyrrhine and catarrhine with an inference of 5 and 6 replacements, respectively, on each stem. The time scale of these 14 replacements is centered on the Eocene and Oligocene, after which the replacement rate slows during the Miocene. Interestingly, this time scale is similar to the time scale of amino acid replacement observed on some subunits of cytochrome *c* oxidase [10]. Ancestral sequence reconstruction using maximum likelihood (ML, Figure S2) confirms results obtained by maximum parsimony (MP). However, the ancestral status of position 44 is ambiguous; indeed, reconstruction using MP proposes a parallel mutation on the catarrhine (A→P) and platyrrhine (A→S) lineages (p = 0.38) but ML results suggest first one mutation (A→P) on the anthropoid stem (p = 0.44) and then the other mutation (A→S) on the platyrrhine lineage. This ambiguity could be due to an ancestral polymorphic state (presence of the two alleles A and P in the ancestral populations) or to a fast mutation rate.

### Evidence for adaptive evolution

Results from the model-based codeml analyses (see Methods) confirmed that *CYCS* omega ratios vary among lineages in primates. Specifically, all the statistical models assuming specific omega values for anthropoid's branches (Model 2a, 2b and 4) are equivalent (ln L of −1315) and significantly better than the one-ratio model (Model 0; ln L of −1324) according to the likelihood ratio test (p<0.001). According to model 1 the omega values (*i.e.*, dN/dS) were anthropoid stem = 0.611, catarrhine stem = 0.821, platyrrhine stem = 0.279 (Figure S2). These values are higher than the other lineages and respectively 8 and 3 times higher than the omega estimated on the M0 tree (0.099). These values, lower than 1.0 (usual threshold for adaptive selection), potentially suggest a release of the selective constraints on some *CYCS* residues. However, all the changes that occurred on anthropoid catarrhine and platyrrhine stems were found conserved in all descendent species (except for residue 44). Because the subsequent conservation of recently changed amino acids is a marker of positive selection [11], the observed value of the omega ratio may result from the combined effects of episodic positive selection on some amino acids and pervasive negative selection on the rest of the protein.

### Primate-replaced residues

In order to evaluate whether the amino replacements are restricted to the anthropoid lineage, we studied the conservation of residues across a taxonomically diverse subset of eukaryotes. Two residues, 44 and 58, have the highest nonsynonymous substitution rate among the sampled eukaryotic species (Table S2). This high evolutionary rate (MAPP value>2.5 and Consurf score >2) could signal weakened purifying selection acting on these residues compared to the others and explain the conservation of these nucleotide substitutions on the root of the catarrhine and platyrrhine lineages. The other residues are moderately to highly conserved (MAPP value<2.5 and Consurf score<2), and residue 83, which was replaced on the anthropoid stem, appears to be the most conserved (MAPP value = 0.48 and Consurf score = −0.58).

### Structural change

**Figure 2:**
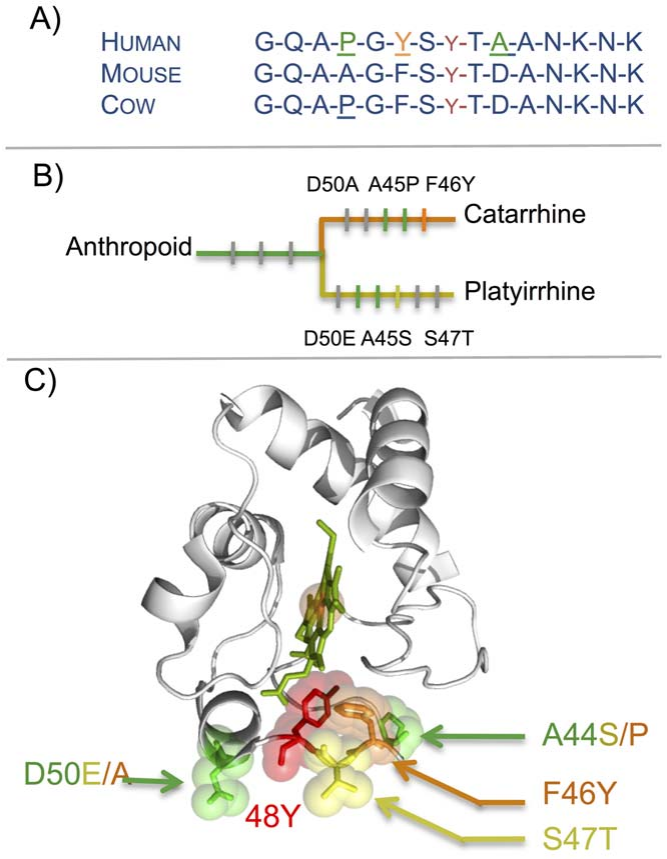
pTYR 48 phosphorylation epitope evolution. A) Sequence alignment of three mammals; residue 48 (in red) has been shown phosphorylated only in cow. The position marked in green has evolved separately on catarrhine and platyrrhine lineages, in orange the position that has evolved only on catarrhini lineages. B) Phylogenic position of evolving amino acid inside pTYR48 epitope. C) Same amino acid shown on the structure of cytochrome *c* (*Bos taurus*, Pdb: 1HRC). The same color code as in 3.a was used. The amino acid that evolved only on the platyrrhini lineage was marked in yellow.

**Figure 3:**
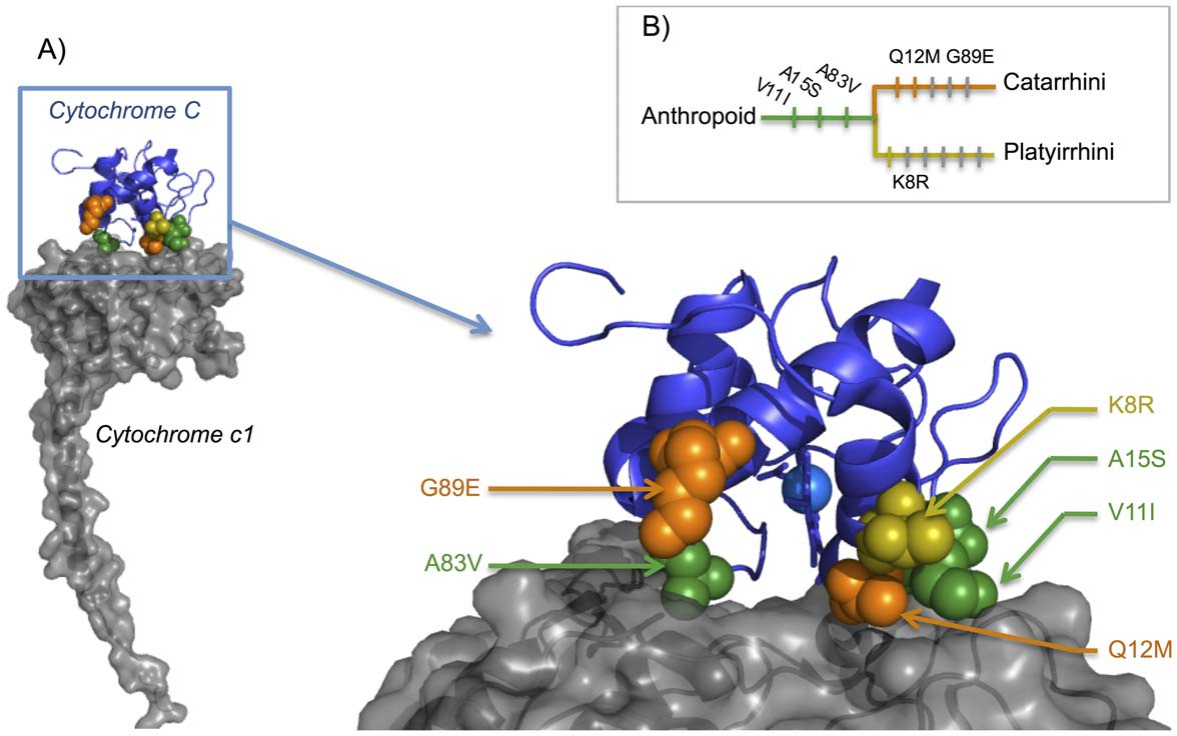
Primate cytochrome *c* complex III binding site evolution. A) Primates with evolving amino acids shown on the structure of cytochrome *c* bound on the cytochrome *c1* (Yeast, Pdb: 3CXH). Mutation on the Anthropoid root are represented in green, Catarrhini in orange, Platyrrhini in yellow. B) Phylogenic position of amino acid replacements potentially acting on the binding site.

Based on current knowledge about cyt *c* we have evaluated which functions have been affected by the amino acid replacements. Only two domains have appeared predominantly affected by the changes (Table S2): (i) **The phosphorylation epitope Tyr-48 (residues 41 to 55**) [12]. Six amino acid replacements were inferred to have occurred independently and in parallel on the platyrrhine and catarrhine lineages. Interestingly, two residues, 44 and 50, were replaced independently in both lineages. Unlike residue 44, which is highly variable among the eukaryotes (Consurf score = 2.5; Table S2), residue 50 has a slow replacement rate among the eukaryotes (Consurf score = 0.9; Table S2), but then is replaced on both lineages. A phylogenetic artifact can be excluded as the cause of this parallel replacement because two different nucleotides distinguish the 50^th^ codon of both lineage from its ancestor (ancestor: GAC→Aspartic acid (PAML p-value = 0.987); platyrrhine : GAA→Glutamic acid ; catarrhine: GCC→Alanine). This pattern could indicate parallel selection forces acting on these two lineages. Indeed, the position 50 appear to be significantly selected according the PAML branch site likelihood test (BEB posterior probability = 0.979, Figure S2) [13]. Furthermore, the changed residues 47 (platyrrhine) and 46 (catarrhine) were both next to tyrosine 48 in the 3D structure (Figure 2) and conserved among the eukaryotes (Consurf score = 0.57 and 0.28, respectively), with significant volume constraint for residue 46 (p-value = 0.01; Table S2). All these changes on the phosphorylation epitope could be produced by a positive evolution pressure on the two lineages affecting phosphorylation of cyt c, although it is not currently possible to conclude to what effect. (ii) **Six replaced residues are located inside or near the binding domains with complex III and IV** (Table S2, Figure 3). These changes could balance or interact with the COX amino acid replacements on the cyt *c* binding site shown in previous studies [9], [14]. The potentially most important changes seem to be A83V and Q12M because these two residues are both conserved among the eukaryotes and both replacements have high potential impact on the molecule (Methods; Table S2). The replacement A83V is evaluated as significantly pathogenous using MAPP (p-value = 0.004) and SIFT (p-value = 0.03); the replacement Q12M presents a borderline p-value (MAPP p-value = 0.09, and SIFT p-value = 0.07). Inversely, position 12 appears significantly selected using the PAML branch site likelihood test (BEB posterior probability = 0.994) [15] and the value for position 83 is not significant (*i.e.*, posterior probability = 0.840). These two changes increase the hydrophobicity of the residues. This binding site evolution could explain the previous result of van Kuilenburg *et al.* who had surprisingly found that anthropoid cyt *c* has a higher affinity for the bovine oxidase than does non-primate cyt *c* [16] .

### Electrostatic evolution

**Figure 4:**
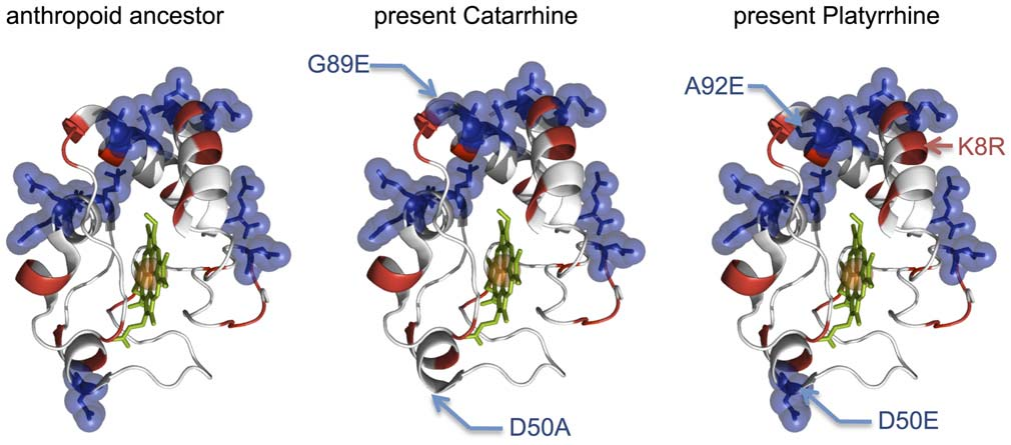
Electrostatic evolution of primate cytochrome *c*. The replacement of charged amino acids on the anthropoid lineage has been shown on the structure of cyt *c* (*Bos taurus*, Pdb: 1HRC). Negatively charged amino acids (D and E) are represented by a blue sphere and positively charged amino acids (K,R,H) are red.

Since electrostatic residues comprising the cyt *c* binding site on COX were subject to adaptive evolution on the anthropoid stem [9], we examined similar cyt *c* evolution. We found a shift in the charge distribution in the catarrhine stem, but without any change in the total charge, and a gain of one negative residue (A92E) in the platyrrhine stem (Figure 4). This doesn't compensate for the neutralization of the cyt c binding site on COX observed by Schmidt *et al.* [9].

### Cytochrome *c* testis isoform

Mouse chromosomal region 2qC3 contains the testis cyt *c* gene (Cyct), and it was possible to localize a putatively orthologous region on human chromosome 2q31 The alignment of both sequences showed a nonsense mutation CGA→TGA replacing the mouse amino acid R by a STOP codon at position 49 (chr2:178,969,083 Human genome [GRCh37/hg19]). The alignment of the same cyt *c* sequences of 12 primates and 3 non primates revealed the existence of this stop codon in all primate sequences but absence on non-primate sequences, suggesting the appearance of this substitution in early primates (Figure S3, GenBank Accession numbers: JF919285-JF919295).

The chromosomal comparison of 3 Mbp surrounding the human somatic cyt *c* gene (7p15) with the human testis cyt *c* pseudogene (2q31) revealed many pairs of paralogous genes: DFNB59/DFNA5, OSBPL6/OSBPL3, testisCyCt/somaticCYCs,NFE2L2/NFE2L3, HNRNPA3/HNRNPA2B1, HOXA/HOXD (Figure S4). This gene organization suggested the duplication of a large chromosomal region. The chromosomal comparison of 3 Mbp surrounding cyt *c* in the chicken genome revealed the existence of the same two orthologous regions (Figure S4). This result suggested that the duplication event is older than the most recent common ancestor of mammals and birds, *i.e*, amniotes ancestor older than 300 Mya [17] .

## Discussion

Our study reveals and dates three major events in the evolutionary history of cytochromes *c*: (i) gene duplication older than 300 Mya, (ii) silencing of the testis isoform 65 Mya (Figures 1 and S3), and (iii) fast and parallel evolution of complex III and IV binding residues of cyt *c* (40-25 Mya; Figs. 3 and 4). We were able to reveal these events because of the extensive phylogeny of primate cytochromes *c* that we carried out based on CYCS sequences of 56 primate and 4 non-primates species, and we will address each of these events below.

In the present study all major groups of primates (*e.g.*, apes, platyrrhines, tarsiers) were recovered with maximal branch support. Interestingly, tarsiers appear as a sister group of anthropoids (Haplorrhini hypothesis), which is consistent with the current view of primate phylogeny [18]. How New World monkey families (*e.g.*, capuchin monkeys, spider monkeys, titi monkeys) are related to each other is still an open question; our results support Pithecidae as a sister group of a clade containing Cebidae and Atelidae [19].

### Duplication of cytochrome *c*

We show that cytochrome *c* belongs with DFN, OSBPL, NFE2L, HNRNPA and the Hox cluster to a chromosomal segment that was duplicated before the most recent common ancestor of mammals and birds (and therefore some reptiles as well). Previous studies have proposed a quadruplication of Hox clusters in early vertebrate evolution [20]. This quadruplication event was recently shown to involve not only Hox but also associated linked genes (the “Hox paralogon”: DFN, OSBPL, NFE2L) and a large chromosomal region duplication was proposed [21], [22]. Because cyt *c* is inside this chromosomal region, we suggest that cyt *c* was also quadruplicated during early vertebrate evolution.

Many genes have been lost in these families after the quadruplication; the two other CYC copies seem to have disappeared or been included in the dozens of CYC pseudogenes present in the human genome [23]. We show here that the same two isoforms are still present in bird (chickens) and mammal (cow and mouse) lineages. In mammals, one is expressed ubiquitously, the other developed tissue specific expression in testis [4] with the known specializations of higher activity in destroying reactive oxygen species and in triggering apoptosis [24]. Interestingly, the gene Pde11a, which has integrated into the first exon of testis cyt *c* in one of its splicing forms (Pde11a3), is also expressed specifically in testis [25]. Thus, the same DNA sequence codes for both proteins but with a different reading frame, potentially stimulating some interesting features of competition and co-evolution between both proteins.

### Silencing of the testis isoform

Sequencing of the Cyct isoform has shown that a nonsense mutation occurred in early primate evolution. This mutation, although nonsense on the Cyct reading frame, is a synonymous mutation in the Pde11a3 reading frame. The phenotypic effect of silencing Cyct can be predicted by studying testis cyt c–null mice. Mice with silenced Cyct produce functional but less efficient sperm and undergo early testicular atrophy, most likely because they don't destroy ROS as well [26]. Therefore, the nonsense mutation on Cyct should decrease reproductive system efficiency and is co-occurring with the decrease of reproductive rate observed in primates compared to rodents and other mammals (AnAge database [27], Figure S5).

### Fast evolution and parallel amino-acid replacements of the somatic isoform

The study of somatic cyt *c* has shown that the difference between human and mouse cyt *c* is due to an accumulation of three amino acid replacements on the anthropoid stem and five on the early catarrhine branch. We report also an accumulation of six amino acid replacements on the early platyrrhine branch.

This rapid evolution of cyt c in two sister clades could be considered as a rapid divergent evolution because 9 amino acids distinguish the platyrrhine's most recent cyt c ancestor from the catarrhine's most recent cyt c ancestor. However, biochemical results have shown kinetic similarity between catarrhine and platyrrhine cyt c [6], [16], suggesting parallel evolution (*i.e.*, the development of a similar trait in related species descending from the same ancestor). The hypothesis of parallel evolution is supported by the observation that the substitutions occurred at similar positions on each lineage, the OXPHOS complex binding site and on a phosphorylation epitope. In addition, rapid amino-acid accumulation begins on the OXPHOS complex binding site on the anthropoid stem, suggesting the same selective pressure is acting during the period before and after lineage separation.

### Selective pressures acting on somatic cyt *c*

Linking the observed primate specific evolution of somatic cyt *c* to the silencing of the testis isoform is problematic because (i) the somatic mutations occur on OXPHOS positions and not on positions implicated in functions specifically different between the two isoforms, apoptotsis and ROS scavenging (Table S2) [28]; (ii) strepsirrhine cyt *c* did not evolve like anthropoid cyt *c* after the silencing of the testis isoform, and (iii) there is not a clear cause and effect since at least 4 My separates the testis isoform silencing from the first mutations on the somatic isoform (Figure 1).

We propose instead that the evolution rate is due to other selective pressures that have acted principally in the Eocene and Oligocene on the early anthropoids but not on other primates. We observe here that evolutionary changes to cyt *c* occur at positions important for respiration and not for apoptosis. This is supported by studies using human or other anthropoid cyt *c* in a reaction with horse or bovine cytochrome oxidase [7] that show both the reactivity and the affinity of anthropoid cyt *c* differs from the non-anthropoid electron carrier. This interpretation is also consistent with previous phylogenetic studies that showed evolution of the OXPHOS system during primate and particularly anthropoid evolution [5], [29], [30], specifically complex III [31] and complex IV [10], [32], [33], [34], [35], [36], [37]. These results particularly complement the previous finding of rapid electrostatic evolution at the binding site of cyt *c* on cytochrome *c* oxidase in anthropoid primates [9].

Our data show also a clear parallel evolution of the tyrosine 48 phosphorylation epitope. It has been shown that phosphorylation at this site decreases respiratory chain activity and is specific to liver [12]. Because the respiratory chain plays a key role in liver function, such as in blood glucose homeostasis and brain feeding (during fasting), these mutations may influence global metabolism, particularly body and brain size (Figure S5).

Finally primate cyt c evolution appears to be clearly more related to respiratory chain evolution than to the loss of testis cyt c. In previous work, we have suggested an influence of respiratory chain evolution on the increase of body and brain size in anthropoid primates. Interestingly, a recent fossil study that suggested body and brain have evolved in both platyrrhine and catarrhine stems [18] is coherent with the parallel evolution for cyt c observed here. This parallel evolution of the respiratory chain on both lineages may have been stimulated by the increase of atmospheric oxygen during the Eocene (20 to 25%) [38], [39], setting the stage for supplying adequate oxygen to a larger organism with a larger brain.

## Methods

### Phylogenetic study

We obtained and compared the DNA sequence of somatic cyt *c* from 56 primate and 4 non-primate species (Datasets S1, S2). Based on somatic cyt *c*, we have estimated phylogenetic relationships among species using maximum likelihood (model chosen using modeltest: GTR+G+I and tree-bisection-reconnection algorithm), maximum parsimony (TBR, all characters having equal weight), and Bayesian approaches (Model: GTR; Base Frequencies: Estimated; Base Heterogeneity = True; No of patterns = 4; No of rates = 4). The tree was rooted using the dog cyt *c* sequence as outgroup. We have estimated divergence times for the whole group using a Bayesian local molecular clock approach [40], [41]. Eight rounds of analysis were performed with different sets of fossils (Table S1). To check the consistency of the results, we ran the analysis three times using multidivtime software (MCMC parameters: samfreq = 5; burnin = 2,000,000; numsamps = 200,000) [42]. For each node, an averaged age associated with a standard deviation was estimated based on the average age obtained for this node by each round.

### Selection analysis

The PAML 3.15 package [43] was used to investigate the signature of positive selection among specific lineages and sites. To investigate whether rate heterogeneity exists among lineages, we compared a one omega (dN/dS) model (M0) with several other models (M1: free ratio model, which allows rates to vary freely among the branches; M2a: two omega ratios were specified, one for all anthropoids (as a total group) and another for the remaining branches in the tree; M2b: three omega ratios were specified, one for the anthropoid stem, another for catarrhini and platyrrhini stems, and the third for the remaining branches; M4: four omega ratios were specified: one for the anthropoid stem, one for catarrhini, one for platyrrhini, one for the remaining branches). We also applied a branch-site test of positive selection on the anthropoid clade as described [13] (Figure S2).

### Amino acid evolution

We have determined amino-acid replacement events during primate evolution and studied the variability of the mutated residues among eukaryotic species. Ancestral sequence reconstruction for each ancestral node was performed (ii) using maximum parsimony (All characters had equal weight and the Character-state optimization used is Delayed transformation) and (ii) using maximum likelihood (PAML 3.15). The evolution rates of the mutated residues have been estimated among the eukaryotic species by two independent phylogenetic programs, Consurf-DB [44] (125 sequences), and MAPP [45] (146 sequences), using default parameters (Table S2). The MAPP program was also used in order to study the amino acid diversity of each residue in eukaryotic evolution and determine the physicochemical constraints (Table S2).

### Functional change

The potential function of each mutated amino acid was assessed based on structural and functional literature [12], [28], [46], [47], [48], [49], [50]. Based on these studies for each function, the residues of cyt *c* were categorized as IN the function or NEAR when the residue is adjacent to a residue inside the function (Table S2). The physicochemical effects of the amino acid change on the mutated residue are issued from MAPP data. The global impact of each non-synonymous mutation on the cyt *c* sequences of the “anthropoid ancestor” is measured independently by the above software [45] using default parameters (Table S2).

### Electrostatically Significant Residue Positions and ES Changes

The gain or loss of any ES residue was studied for each primate lineage, as well as the change of charge distribution among the lineages. A positively (Arg or Lys) or negatively (Asp or Glu) charged residue was treated as an ES position.

### Testis isoform

We obtained and compared the DNA sequence of the testis cyt *c* locus of 12 primate and 3 non-primate species (Figure S3, Dataset S1). A chromosomal comparison of 3 Mbp surrounding the human somatic cyt *c* gene and the testis cyt *c* pseudogene was performed [51]. In order to know if the duplication of the cyt *c* gene is older than mammals, we checked the existence of both cyt *c* isoforms in birds by BLAT Search [52] of chicken (*Gallus gallus*) [51] and performed a tblastn analysis on all genes bordering chicken cyt *c* for 3 Mb against the human genome.

## Supporting Information

Dataset S1 **List of primer used for amplification and sequencing.**
(XLS)

Dataset S2 **Cytochrome** ***c*** **gene sequences for 56 primate and 4 non-primate species.**
(TXT)

Figure S1 **Bootstrap and posterior Bayes probability for each branch of the tree.**
(PPTX)

Figure S2 **Result of the PAML analysis regarding the signature of positive selection among specific lineages and sites.**
(DOCX)

Figure S3 **Nonsense mutation on testis cytochrome** ***c*** **CGA/TGA.**
(PPTX)

Figure S4 **Comparison of human and chicken chromosomal region bordering cytochrome** ***c*** **sequence.** Genes belonging to the duplicated paralogon have been colored.
(PPTX)

Figure S5 **Life traits of primates and rodents from AnAge database (** http://genomics.senescence.info/species/ **).** Body mass and litter sizes are the average of all available data by phylogenetic group. Longevity is the maximum lifespan observed in each group.
(PPTX)

Table S1 **Age estimated using multidivtime software for each round with different sets of fossils.**
(XLSX)

Table S2 **Characterization of cyt c amino-acid replacements.**
(DOC)
