# Supplementary material for: Silencing, Positive Selection and Parallel Evolution: Busy History of Primate Cytochromes c
Source: PLoS One. 2011 Oct 18;6(10):e26269. doi: 10.1371/journal.pone.0026269 (PMC3196546; doi:10.1371/journal.pone.0026269)
Supplement: Figure S1 — Bootstrap and posterior Bayes probability for each branch of the tree. (PPTX) [file pone.0026269.s003.pptx]

## Slide 1
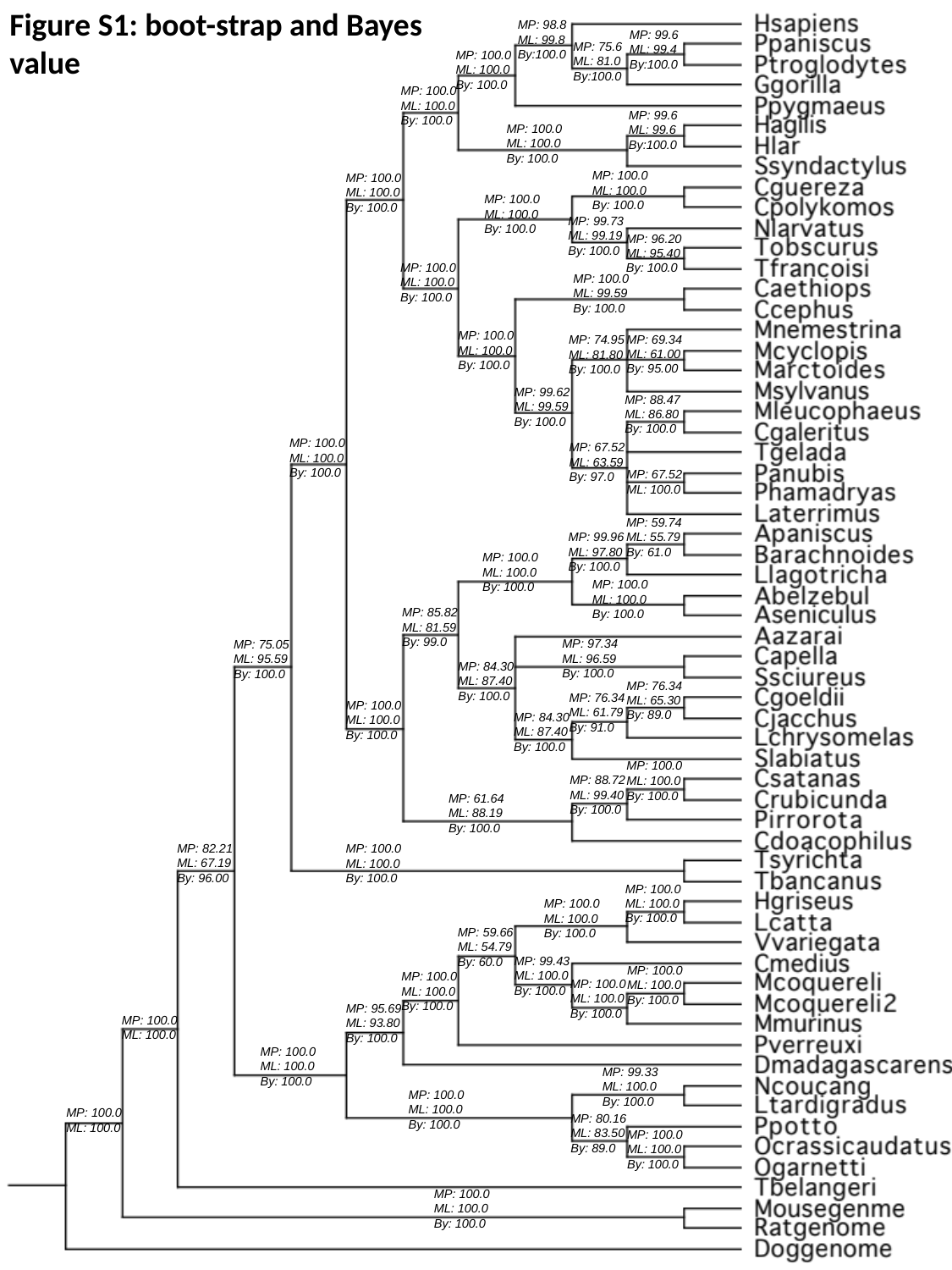

Figure S1: boot-strap and Bayes value
MP: 98.8
ML: 99.8
By:100.0
MP: 99.6
ML: 99.4 By:100.0
MP: 75.6
ML: 81.0
By:100.0
MP: 100.0
ML: 100.0
By: 100.0
MP: 100.0
ML: 100.0
By: 100.0
MP: 99.6
ML: 99.6
By:100.0
MP: 100.0
ML: 100.0
By: 100.0
MP: 100.0
ML: 100.0
By: 100.0
MP: 100.0
ML: 100.0
By: 100.0
MP: 100.0
ML: 100.0
By: 100.0
MP: 99.73
ML: 99.19
By: 100.0
MP: 96.20
ML: 95.40
By: 100.0
MP: 100.0
ML: 100.0
By: 100.0
MP: 100.0
ML: 99.59
By: 100.0
MP: 100.0
ML: 100.0
By: 100.0
MP: 69.34
ML: 61.00
By: 95.00
MP: 74.95
ML: 81.80
By: 100.0
MP: 99.62
ML: 99.59
By: 100.0
MP: 88.47
ML: 86.80
By: 100.0
MP: 100.0
ML: 100.0
By: 100.0
MP: 67.52
ML: 63.59
By: 97.0
MP: 67.52
ML: 100.0
MP: 59.74
ML: 55.79
By: 61.0
MP: 99.96
ML: 97.80
By: 100.0
MP: 100.0
ML: 100.0
By: 100.0
MP: 100.0
ML: 100.0
By: 100.0
MP: 85.82
ML: 81.59
By: 99.0
MP: 97.34
ML: 96.59
By: 100.0
MP: 75.05
ML: 95.59
By: 100.0
MP: 84.30
ML: 87.40
By: 100.0
MP: 76.34
ML: 65.30
By: 89.0
MP: 76.34
ML: 61.79
By: 91.0
MP: 100.0
ML: 100.0
By: 100.0
MP: 84.30
ML: 87.40
By: 100.0
MP: 100.0
ML: 100.0
By: 100.0
MP: 88.72
ML: 99.40
By: 100.0
MP: 61.64
ML: 88.19
By: 100.0
MP: 82.21
ML: 67.19
By: 96.00
MP: 100.0
ML: 100.0
By: 100.0
MP: 100.0
ML: 100.0
By: 100.0
MP: 100.0
ML: 100.0
By: 100.0
MP: 59.66
ML: 54.79
By: 60.0
MP: 99.43
ML: 100.0
By: 100.0
MP: 100.0
ML: 100.0
By: 100.0
MP: 100.0
ML: 100.0
By: 100.0
MP: 100.0
ML: 100.0
By: 100.0
MP: 95.69
ML: 93.80
By: 100.0
MP: 100.0
ML: 100.0
MP: 100.0
ML: 100.0
By: 100.0
MP: 99.33
ML: 100.0
By: 100.0
MP: 100.0
ML: 100.0
By: 100.0
MP: 100.0
ML: 100.0
MP: 80.16
ML: 83.50
By: 89.0
MP: 100.0
ML: 100.0
By: 100.0
MP: 100.0
ML: 100.0
By: 100.0
