# Supplementary material for: Silencing, Positive Selection and Parallel Evolution: Busy History of Primate Cytochromes c
Source: PLoS One. 2011 Oct 18;6(10):e26269. doi: 10.1371/journal.pone.0026269 (PMC3196546; doi:10.1371/journal.pone.0026269)
Supplement: Figure S2 — Result of the PAML analysis regarding the signature of positive selection among specific lineages and sites. (DOCX) [file pone.0026269.s004.docx]

**Figure S2:**

PAML dS/dN

CODEML ANALYSES MODEL test

**PAML BRANCH SITE TEST**


ML ANCESTRAL RECONSTRUCTION
