# Supplementary material for: Silencing, Positive Selection and Parallel Evolution: Busy History of Primate Cytochromes c
Source: PLoS One. 2011 Oct 18;6(10):e26269. doi: 10.1371/journal.pone.0026269 (PMC3196546; doi:10.1371/journal.pone.0026269)
Supplement: Figure S3 — Nonsense mutation on testis cytochrome c CGA/TGA. (PPTX) [file pone.0026269.s005.pptx]

## Slide 1
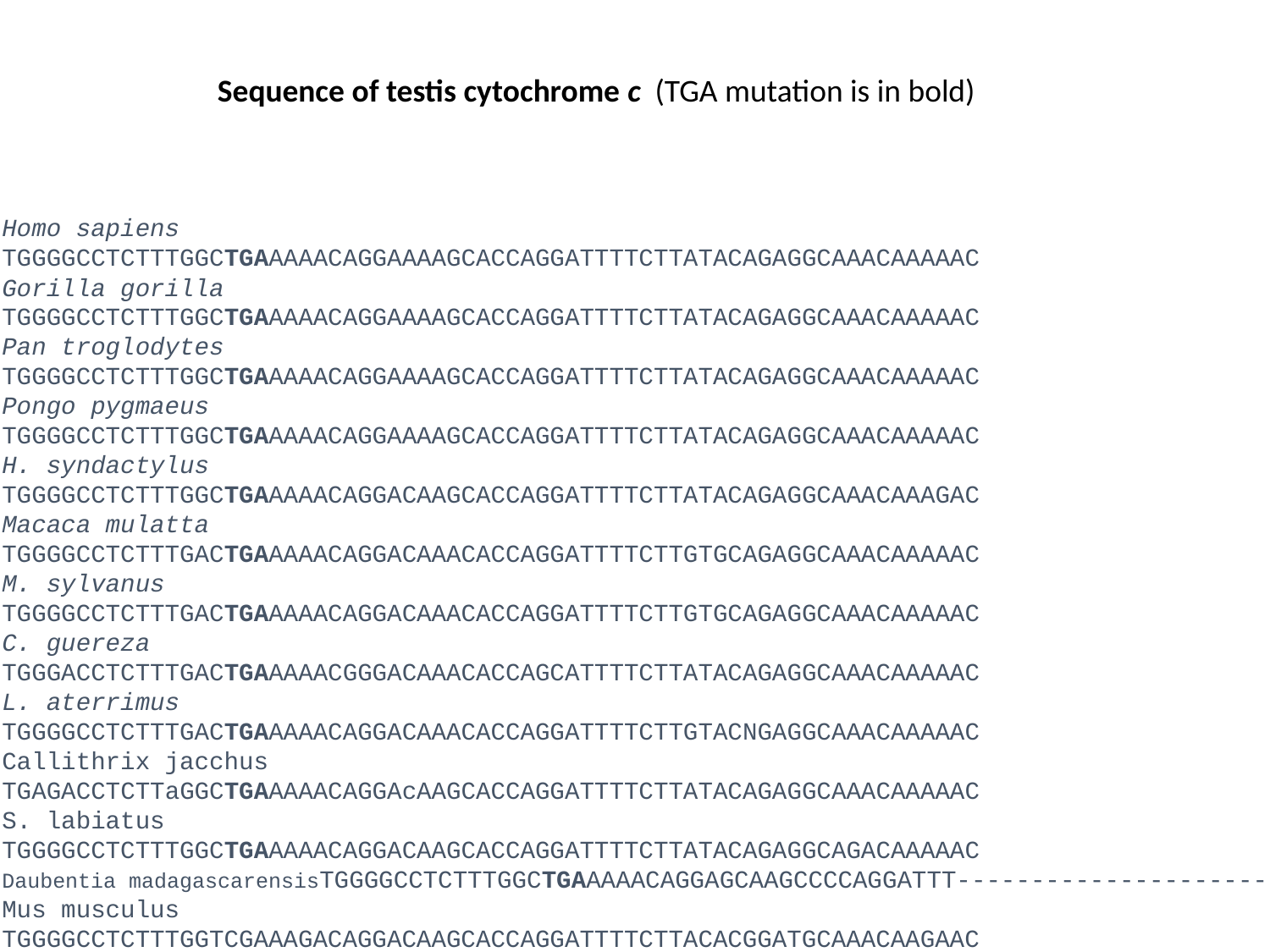

Sequence of testis cytochrome c (TGA mutation is in bold)
Homo sapiens 		TGGGGCCTCTTTGGCTGAAAAACAGGAAAAGCACCAGGATTTTCTTATACAGAGGCAAACAAAAAC
Gorilla gorilla 		TGGGGCCTCTTTGGCTGAAAAACAGGAAAAGCACCAGGATTTTCTTATACAGAGGCAAACAAAAAC
Pan troglodytes 		TGGGGCCTCTTTGGCTGAAAAACAGGAAAAGCACCAGGATTTTCTTATACAGAGGCAAACAAAAAC
Pongo pygmaeus 		TGGGGCCTCTTTGGCTGAAAAACAGGAAAAGCACCAGGATTTTCTTATACAGAGGCAAACAAAAAC
H. syndactylus		tggggcctctttggctgaaaaacaggacaagcaccaggattttcttatacagaggcaaacaaagac
Macaca mulatta 		TGGGGCCTCTTTGACTGAAAAACAGGACAAACACCAGGATTTTCTTGTGCAGAGGCAAACAAAAAC
M. sylvanus			tggggcctctttgactgaaaaacaggacaaacaccaggattttcttgtgcagaggcaaacaaaaac
C. guereza			tgggacctctttgactgaaaaacgggacaaacaccagcattttcttatacagaggcaaacaaaaac
L. aterrimus			tggggcctctttgactgaaaaacaggacaaacaccaggattttcttgtacNgaggcaaacaaaaac
Callithrix jacchus	tgagaCCTCTTaGGCTGAAAAACAGGAcAAGCACCAGGATTTTCTTATACAGAGGCAAACAAAAAC
S. labiatus			tggggcctctttggctgaaaaacaggacaagcaccaggattttcttatacagaggcagacaaaaac
Daubentia madagascarensisTggggcctctttggctgaaaaacaggagcaagccccaggattt---------------------
Mus musculus 		TGGGGCCTCTTTGGTCGAAAGACAGGACAAGCACCAGGATTTTCTTACACGGATGCAAACAAGAAC
Rattus norvegicus 	TGGGGCCTTTTTGGCCGAAAGACTGGACAAGCACCAGGATTTTCTTACACGGATGCAAACAAGAAC
Equus caballus 		TGGGGCCTTTTTGGCCGAAAAACAGGACAAGCACCAGGATTTTCTTACTCTGATGCAAACAAAAAC
