# Supplementary material for: Silencing, Positive Selection and Parallel Evolution: Busy History of Primate Cytochromes c
Source: PLoS One. 2011 Oct 18;6(10):e26269. doi: 10.1371/journal.pone.0026269 (PMC3196546; doi:10.1371/journal.pone.0026269)
Supplement: Figure S4 — Comparison of human and chicken chromosomal region bordering cytochrome c sequence. Genes belonging to the duplicated paralogon have been colored. (PPTX) [file pone.0026269.s006.pptx]

## Slide 1
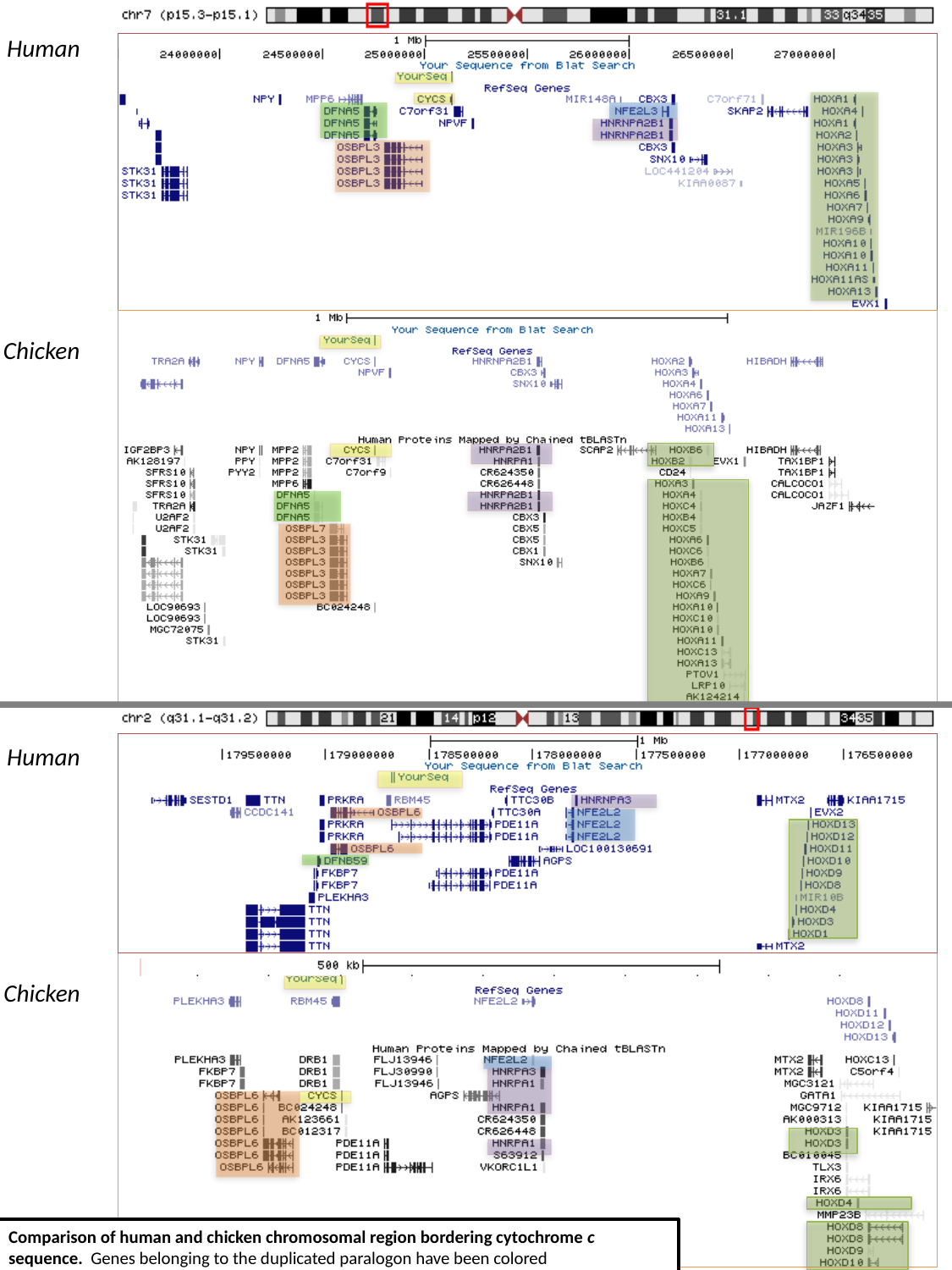

Human
Chicken
Human
Chicken
Comparison of human and chicken chromosomal region bordering cytochrome c sequence. Genes belonging to the duplicated paralogon have been colored
