# Supplementary material for: Silencing, Positive Selection and Parallel Evolution: Busy History of Primate Cytochromes c
Source: PLoS One. 2011 Oct 18;6(10):e26269. doi: 10.1371/journal.pone.0026269 (PMC3196546; doi:10.1371/journal.pone.0026269)
Supplement: Figure S5 — Life traits of primates and rodents from AnAge database ( http://genomics.senescence.info/species/ ). Body mass and litter sizes are the average of all available data by phylogenetic group. Longevity is the maximum lifespan observed in each group. (PPTX) [file pone.0026269.s007.pptx]

## Slide 1
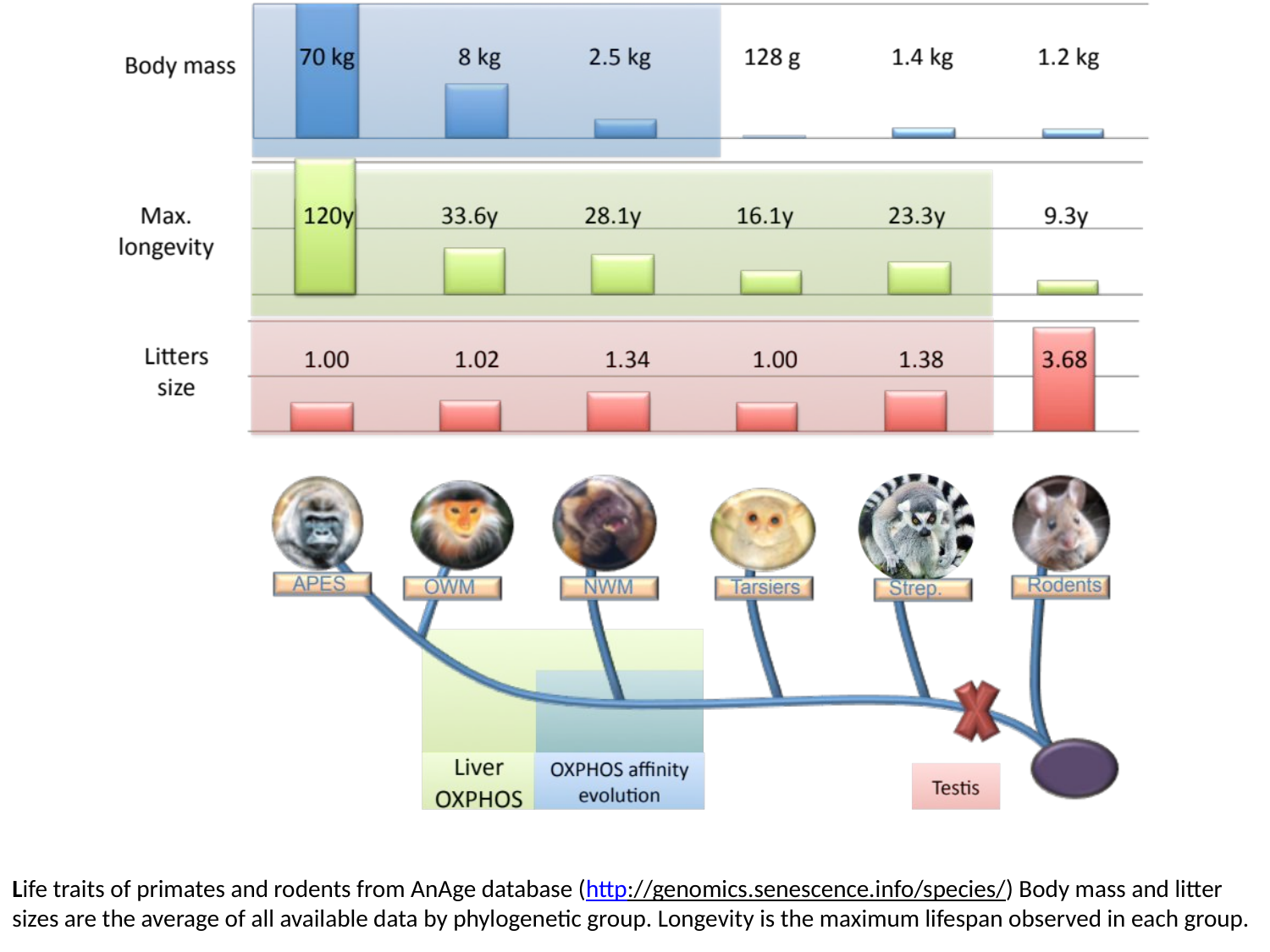

Life traits of primates and rodents from AnAge database (http://genomics.senescence.info/species/) Body mass and litter sizes are the average of all available data by phylogenetic group. Longevity is the maximum lifespan observed in each group.
