# Supplementary material for: Silencing, Positive Selection and Parallel Evolution: Busy History of Primate Cytochromes c
Source: PLoS One. 2011 Oct 18;6(10):e26269. doi: 10.1371/journal.pone.0026269 (PMC3196546; doi:10.1371/journal.pone.0026269)
Supplement: Table S2 — Characterization of cyt c amino-acid replacements. (DOC) [file pone.0026269.s009.doc]

|  | **>Anthropoid** | | | | | **>>Catarrhini** | | | | | | | | | | | **>>>**  **Apes** | | **>>Platyrrhini** | | | | | | | | **>>>**  **NW1** |
| --- | --- | --- | --- | --- | --- | --- | --- | --- | --- | --- | --- | --- | --- | --- | --- | --- | --- | --- | --- | --- | --- | --- | --- | --- | --- | --- | --- |
|  | V11I | | A15S | | A83V | Q12M | | A44P | | | F46Y | | D50A | | | G89E | T58I | | K8R | A44S | S47T | | D50E | T58I | | A92E | L35I |
| **RESIDUE FUNCTION** | | | | | | | | | | | | | | | | | | | | | | | | | | | |
| Axial ligands of heme iron | Ø | | Ø | | Ø | Ø | | Ø | | | Ø | | Ø | | | Ø | Ø | | Ø | Ø | Ø | | Ø | Ø | | Ø | Ø |
| Binding of lipids | Ø | | Ø | | Ø | Ø | | Ø | | | Ø | | Ø | | | Ø | Ø | | Ø | Ø | Ø | | Ø | Ø | | near | Ø |
| Hydrophobic crevice | Ø | | Ø | | IN | Ø | | Ø | | | Ø | | Ø | | | Ø | Ø | | Ø | Ø | Ø | | Ø | Ø | | Ø | Ø |
| APAF binding site | Ø | | Ø | | Ø | Ø | | Ø | | | Ø | | Ø | | | Ø | Ø | | near | Ø | Ø | | Ø | Ø | | Ø | Ø |
| ATP binding site 1 | Ø | | Ø | | Ø | Ø | | Ø | | | Ø | | Ø | | | near | Ø | | Ø | Ø | Ø | | Ø | Ø | | near | Ø |
| ATP binding site 2 | Ø | | Ø | | Ø | Ø | | Ø | | | Ø | | Ø | | | Ø | Ø | | Ø | Ø | Ø | | Ø | Ø | | Ø | Ø |
| ATP binding site 3 | Ø | | Ø | | Ø | near | | Ø | | | Ø | | Ø | | | Ø | Ø | | Ø | Ø | Ø | | Ø | Ø | | Ø | Ø |
| Phosphorylation epitope Tyr 97 | Ø | | Ø | | Ø | Ø | | Ø | | | Ø | | Ø | | | Ø | Ø | | Ø | Ø | Ø | | Ø | Ø | | near | Ø |
| Phosphorylation epitope Tyr 48 | Ø | | Ø | | Ø | Ø | | IN | | | IN | | IN | | | Ø | Ø | | Ø | IN | IN | | IN | Ø | | Ø | Ø |
| Complex III binding site | IN | | near | | IN | IN | | Ø | | | Ø | | Ø | | | IN | Ø | | Ø | Ø | Ø | | Ø | Ø | | Ø | Ø |
| Complex IV binding site | near | | near | | IN | IN | | Ø | | | Ø | | IN | | | Ø | Ø | | IN | Ø | Ø | | Ø | Ø | | Ø | Ø |
| **RESIDUE VARIATION AND EVOLUTION AMONG EUKARYOTES** | | | | | | | | | | | | | | | | | | | | | | | | | | | |
| 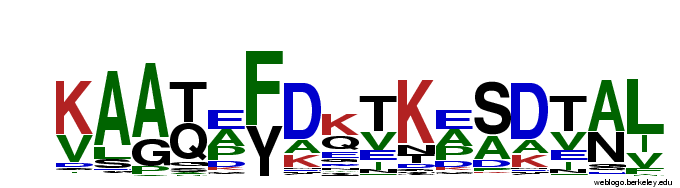 | |  | | | | | | | | | | | | | | | | | | | | | | | | | |
| Consurf score | | 0.61 | | 0.75 | -0.58 | | -0.22 | | | 2.49 | | 0.57 | | 0.90 | 1.90 | | 2.44 | 0.39 | | 2.49 | 0.28 | 0.90 | | 2.44 | 0.34 | | 0.27 |
| MAPP Evolutionary rate | | 1.09 | | 1.27 | 0.48 | | 0.97 | | | 2.85 | | 0.91 | | 1.57 | 2.24 | | 3.09 | 0.97 | | 2.85 | 1.27 | 1.57 | | 3.08 | 1.94 | | 1.39 |
| Hydropathy constraint  (MAPP p-value) | | 1.00 | | 1.00 | 1.00 | | 1.00 | | | 1.00 | | 1.00 | | 1.00 | 0.92 | | 1.00 | 0.67 | | 1.00 | 1.00 | 1.00 | | 1.00 | 1.00 | | **0.02** |
| Polarity constraint  (MAPP p-value) | | 1.00 | | 0.99 | **0.19** | | 1.00 | | | 1.00 | | 0.99 | | 1.00 | 1.00 | | 1.00 | 0.99 | | 1.00 | 1.00 | 1.00 | | 1.00 | 1.00 | | **0.02** |
| Charge  constraint  (MAPP p-value) | | 1.00 | | 1.00 | 1.00 | | 1.00 | | | 1.00 | | 1.00 | | 1.00 | 1.00 | | 1.00 | 1.00 | | 1.00 | 1.00 | 1.00 | | 1.00 | 1.00 | | 1.00 |
| Volume constraint  (MAPP p-value) | | 1.00 | | 0.99 | **0.28** | | 1.00 | | | 1.00 | | **0.01** | | 1.00 | 1.00 | | 1.00 | 1.00 | | 1.00 | **0.38** | 1.00 | | 1.00 | 0.87 | | 0.93 |
| **MAPP VALUE OF PRIMATE RESIDUE PARAMETERS EVOLUTION** | | | | | | | | | | | | | | | | | | | | | | | | | | | |
| **Hydropathy** ancestor | | 2.22 | | 1.16 | 1.16 | -1.17 | | | 1.16 | | | 1.60 | | -1.17 | 0.2 | | 0.06 | -1.34 | | 1.16 | 0.02 | -1.17 | | 0.06 | 1.16 | | 2.04 |
| **Hydropathy** present | | 2.35 | | 0.02 | 2.22 | 1.21 | | | -0.33 | | | -0.2 | | 1.16 | -1.17 | | 2.35 | -1.61 | | 0.02 | 0.06 | -1.17 | | 2.35 | -1.17 | | 2.35 |
| **Polarity** ancestor | | 1.05 | | 0.85 | 0.85 | -0.28 | | | 0.85 | | | 1.27 | | -1.29 | 0.73 | | 0.77 | -1.21 | | 0.85 | 0.65 | -1.29 | | 0.77 | 0.85 | | 1.09 |
| **Polarity** present | | 1.15 | | 0.65 | 1.05 | 1.21 | | | 0.5 | | | 0.4 | | 0.85 | -1.09 | | 1.15 | -1.91 | | 0.65 | 0.77 | -1.09 | | 1.15 | -1.09 | | 1.15 |
| **Side-chain vol.** ancestor | | 3.97 | | -0.79 | -0.79 | 4.32 | | | -0.79 | | | 8.59 | | 1.3 | -3.43 | | 1.76 | 6.62 | | -0.79 | -0.75 | 1.3 | | 1.76 | -0.79 | | 6.44 |
| **Side-chain vol.** present | | 6.44 | | -0.75 | 3.97 | 6.09 | | | 1.44 | | | 8.93 | | -0.79 | 3.82 | | 6.44 | 7.06 | | -0.75 | 1.76 | 3.82 | | 6.44 | 3.82 | | 6.44 |
| GLOBAL IMPACT SCORE | | | | | | | | | | | | | | | | | | | | | | | | | | | |
| MAAP SCORE p-value | | 0.82 | | 0.06 | 0.004 | | 0.09 | | | 0.30 | | 0.28 | | 0.11 | 0.51 | | 0.45 | 0.73 | | 0.35 | 0.14 | 0.29 | | 0.45 | 0.01 | | 0.89 |
| SIFT Score | | 0.09 | | 0.14 | 0.03 | | 0.07 | | | 0.49 | | 1.00 | | 0.52 | 0.37 | | 0.39 | 0.10 | | 0.58 | 0.11 | 0.44 | | 0.39 | 0.56 | | 0.25 |

Supplementary information 4 : **Characterization of anthropoid cyt *c* amino-acid replacement**

(i) residue function: Based on studies (1-7). the residues of cyt *c* are categorized as IN the function or NEAR when the residue is bordering another residue inside the function.(ii) residue variation is calculated by consurf-DB and represent using weblogo (<http://weblogo.berkeley.edu/>), residues evolutionary rates of are estimated among the eukaryotic species by 2 Consurf-DB and Mapp.

1. Craig DB & Wallace CJ (1991) The specificity and Kd at physiological ionic strength of an ATP-binding site on cytochrome c suit it to a regulatory role. *Biochem J* 279 ( Pt 3):781-786.

2. Patriarca A*, et al.* (2009) ATP acts as a regulatory effector in modulating structural transitions of cytochrome c: implications for apoptotic activity. *Biochemistry* 48(15):3279-3287.

3. Yu T, Wang X, Purring-Koch C, Wei Y, & McLendon GL (2001) A mutational epitope for cytochrome C binding to the apoptosis protease activation factor-1. *J Biol Chem* 276(16):13034-13038.

4. Kalanxhi E & Wallace CJ (2007) Cytochrome c impaled: investigation of the extended lipid anchorage of a soluble protein to mitochondrial membrane models. *Biochem J* 407(2):179-187.

5. Yu H, Lee I, Salomon AR, Yu K, & Huttemann M (2008) Mammalian liver cytochrome c is tyrosine-48 phosphorylated in vivo, inhibiting mitochondrial respiration. *Biochim Biophys Acta* 1777(7-8):1066-1071.

6. Lee I*, et al.* (2006) New prospects for an old enzyme: mammalian cytochrome c is tyrosine-phosphorylated in vivo. *Biochemistry* 45(30):9121-9128.

7. Solmaz SR & Hunte C (2008) Structure of complex III with bound cytochrome c in reduced state and definition of a minimal core interface for electron transfer. *J Biol Chem* 283(25):17542-17549.
